# Stroke Caused by Vasculitis Induced by Periodontitis-Associated Oral Bacteria after Wisdom Teeth Extraction

**DOI:** 10.3390/brainsci14060550

**Published:** 2024-05-28

**Authors:** David Kiramira, Timo Uphaus, Ahmed Othman, Ralf Heermann, James Deschner, Lena Katharina Müller-Heupt

**Affiliations:** 1 Department of Periodontology and Operative Dentistry, University Medical Center of the Johannes Gutenberg University Mainz, 55131 Mainz, Germany; 2 Department of Neurology and Focus Program Translational Neuroscience (FTN), Rhine Main Neuroscience Network (rmn2), University Medical Center of the Johannes Gutenberg University Mainz, 55131 Mainz, Germany; 3 Department of Neuroradiology, University Medical Center of the Johannes Gutenberg University Mainz, 55131 Mainz, Germany; 4 Institute of Molecular Physiology, Microbiology and Biotechnology, Johannes Gutenberg University Mainz, 55131 Mainz, Germany; 5 Institute for Biotechnology and Drug Research gGmbH (ibwf), 55128 Mainz, Germany

**Keywords:** bacteremia, meningitis, periodontitis, *Porphyromonas gingivalis*, ischemic stroke, vasculitis, quorum sensing, interkingdom signaling

## Abstract

Invasive dental procedures, such as wisdom teeth removal, have been identified as potential triggers for vascular events due to the entry of oral bacteria into the bloodstream, leading to acute vascular inflammation and endothelial dysfunction. This study presents the case of a 27-year-old healthy male who developed ischemic stroke resulting from bacteremia after undergoing wisdom teeth extraction. Initially, the patient experienced fever and malaise, which were followed by right-sided hemiplegia. Diagnostic imaging, including a CT scan, identified a subacute infarction in the posterior crus of the left internal capsule, and MRI findings indicated inflammatory changes in the masticatory muscles. Further investigations involving biopsies of the masticatory muscles, along with blood and cerebrospinal fluid samples, confirmed bacterial meningitis with associated vasculitis. Notably, oral bacteria linked to periodontitis, including *Porphyromonas gingivalis*, *Fusobacterium nucleatum*, *Tannerella forsythia*, and *Parvimonas micra*, were found in the biopsies and microbiological analyses. To the best of our knowledge, this is the first reported case showing that bacteremia following dental procedures can lead to such severe neurological outcomes. This case underscores the importance of recognizing bacteremia-induced vasculitis in patients presenting with neurological symptoms post-dental procedures, emphasizing the broader implications of oral infections in such pathologies.

## 1. Introduction

Bacteremia following dental treatments is a common occurrence [1,2] due to the abundance of bacteria in the oral cavity. While no published case reports have directly established a connection between bacteremia and ischemic stroke, research has suggested a rise in vascular events occurring within four weeks following invasive dental procedures [3] and individuals with inadequate oral hygiene face a higher susceptibility to conditions like stroke [4]. However, a systematic review and meta-analysis found that patients undergoing invasive dental treatments did not show a significant increase in vascular risk within the eight weeks following treatment. Despite this, a statistically insignificant rise in the overall incidence rate for all vascular events was noted between five and eight weeks post-procedure. Notably, this increase was primarily attributed to a statistically significant rise in the incidence rate for ischemic stroke during the same timeframe [5]. Another review examining the association between periodontitis, gingivitis, and stroke consistently revealed a significant correlation between stroke and periodontal disease. The authors suggest that this association may arise from a pro-inflammatory immune response triggered by pathogenic bacteria. These bacteria enter the bloodstream during routine activities such as chewing, brushing, or minor dental procedures, resulting in transient systemic bacteremia. Subsequent endothelial activation may lead to the production of pro-inflammatory cytokines such as IL-6 and interferons, contributing to plaque rupture, platelet aggregation, thrombus formation, and thromboembolism, all of which are contributing factors to stroke [6]. Additionally, there is one reported case of posterior circulation stroke secondary to a dental procedure, attributed to vertebral artery dissection resulting from an extended and rotated neck position for over 1.5 h in the dentist’s chair [7].

Disruption of bacterial biofilms in the gingival tissue niche can result in the spread of micro-organisms into the bloodstream. It is not only invasive oral procedures like tooth extractions but also routine oral hygiene practices such as brushing, flossing, and even natural processes like chewing that can be considered as potential disruptors of the delicate barrier between the oral biofilm and the host tissues [8]. Bacteremia following tooth extraction has been reported to occur in a range from 72.1% to 96.2% [1,9], with a specific rate of 62% after third molar surgery [10]. Additionally, bacteremia was found to occur more frequently when teeth were extracted due to inflammatory dental diseases [1]. It was also reported that subjects with periodontal disease exhibited the highest intensity of bacteremia. When it comes to causing bacteremia, dental extractions, subgingival instrumentation, and oral hygiene procedures stand out with the highest incidence, while toothbrushing, flossing, and chewing result in bacteremia but occur less frequently [2].

The most frequently isolated bacterial genera include *Streptococcus* spp. [1,9] *Lactobacillus*, and *Staphylococcus*, along with the presence of anaerobic bacteria such as *Eubacterium*, *Peptostreptococcus*, and *Propionibacterium* [1]. In cases of third molar surgery, *Streptococcus viridans* was identified as the most common bacterium (87.9%) [10]. In the case of the anaerobic bacteria *Fusobacterium nucleatum* and *Porphyromonas* have been reported to cause brain abscesses. *Fusobacterium nucleatum* [10,11] and *Porphyromonas gingivalis* [12], both anaerobic bacteria and periodontopathogens, have been documented as causative agents of brain abscesses but to our knowledge, no case of stroke or meningitis was reported up to date.

Given the observed significant increase in the occurrence of vascular events within the initial weeks following invasive dental treatment [3], it is crucial to consider the potential role of oral bacteremia in inducing acute inflammation and endothelial dysfunction. This consideration is important both during the treatment of patients in the dental clinic and when monitoring patients after invasive surgical procedures.

## 2. Case Presentation

A 27-year-old man was admitted as emergency to the stroke unit with a right-side paralysis and left temporal headache. The patient exhibited neurological symptoms, including partial facial paralysis on the right side and dysarthria. Additionally, there was a motor deficit characterized by complete paralysis of the right arm and significant weakness in the right leg (muscle strength rated as M2 on the British Medical Research Council Scale [BMRC]). Due to symptom onset before 13 h, intravenous thrombolysis was no longer possible. CT angiography did not show large vessel occlusion, so that no acute recanalization therapy was performed.

**Figure 1:**
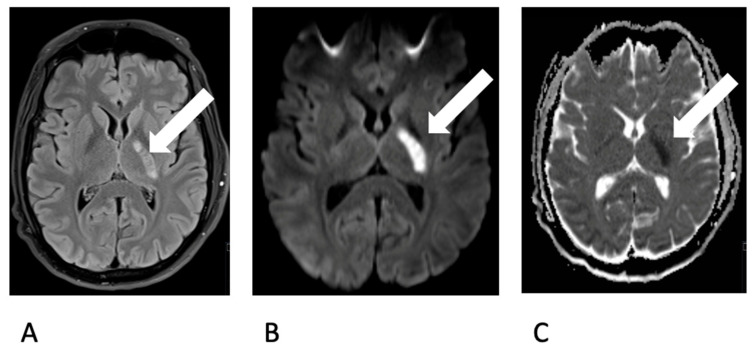
Depiction of an ischemic lesion (white arrow) in the posterior limb of the left internal capsule (**A**. choroidal artery territory) on MRI. Demarcation on T2 FLAIR-w image (**A**) and Diffusion restriction on b1000 image (**B**) and ADC map (**C**).

The previously healthy young man, without any cardiovascular risk factors, had undergone wisdom tooth extraction one week prior. Following the procedure, he developed symptoms such as fever and a general sense of illness. A cranial CT scan was conducted, indicating a demarcated infarct in the posterior crus of the left internal capsule, characterized by hypodensity. A lumbar puncture revealed the presence of lymphomonocytic cells (approximately 800 cells) and elevated lactate levels, consistent with a diagnosis of bacterial meningitis accompanied by vasculitis. Triple therapy with ceftriaxone, ampicillin, and aciclovir was initiated. An MRI scan confirmed CT findings (Figure 1).

**Figure 2:**
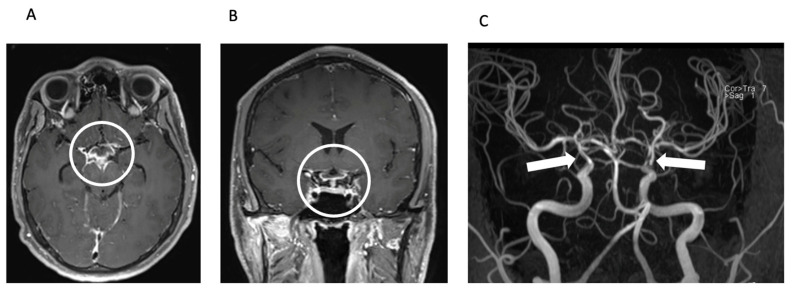
Meningitis and secondary CNS vasculitis on MRI. Axial (**A**) and coronal (**B**) reconstructions of contrast-enhanced T1 SPACE image showing basal contrast enhancement predominantly in the basal cisterns around the circulus of Willis (circle). TOF MR angiography (MIP, posteroanterior view—(**C**)) shows severe stenoses of the intradural proximal cerebral arteries, predominantly in the distal ICA including carotid T bilaterally (arrows).

The radiologist’s observation of intraventricular contrast agent enhancement led to a suspicion of pyogenic meningitis with concomitant vasculitis (Figure 2).

Competing causes of cerebral ischemia were thoroughly excluded. Considering the presence of basal meningitis, a positive quantiferon test, and cerebrospinal fluid (CSF) findings consistent with tubercular meningitis, a quadruple tuberculostatic therapy regimen and cortisone were administered for a few days. However, repeated polymerase chain reaction (PCR) tests for tuberculosis bacteria yielded negative results. Table 1 provides an overview of the blood and CSF tests conducted during the patient’s hospitalization.

**Figure 3:**
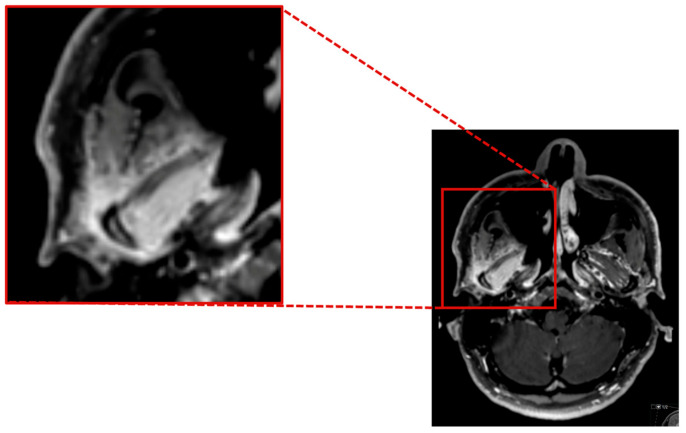
Contrast enhanced axial T1 SPACE showing a marked inflammatory contrast enhancement of the mandible head and the surrounding soft tissue, especially the lateral pterygoidal and masseter muscle.

As the herpes simplex virus and Listeria tests PCR yielded negative results, the administration of aciclovir and ampicillin was discontinued. A follow-up MRI revealed inflammatory changes in the region of the right jaw joint and the masticatory muscles (Figure 3).

Specimens were obtained from the right temporomandibular joint and the right lateral pterygoid muscle for histopathological examination. Furthermore, extensive genetic diagnostics for CSF were performed using Next Generation Sequencing (NGS) analyzing cell-free DNA (Noscendo Pathogen Diagnostics, Noscendo GmbH, Duisburg, Germany), which revealed an infection with periodontopathogens *Fusobacterium nucleatum*, *Porphyromonas gingivalis*, *Tannerella forsythia*, and *Parvimonas micra* (Table 2). The same pathogens in both biopsies—the temporomandibular joint and the muscle samples—were found, confirming their connection to the CSF infection. Antibiotic therapy was modified to ceftriaxone and metronidazole which resulted in a decrease in CSF cell count, lactate, and protein levels. The most probable source of inflammation appears to be an abscess in the region of the temporomandibular joint with oral bacteria associated with periodontitis from the oral cavity following wisdom tooth extraction.

## 3. Discussion

Bacteremia is a well-documented outcome following invasive or noninvasive dental treatments, largely due to the abundant microbial flora in the oral cavity [2,13], and was shown to be influenced by periodontal parameters [14]. The severity of bacteremia tends to be greater in cases involving inflammatory dental conditions [15] and is particularly linked with procedures that disrupt dental or periodontal biofilms, including extractions and deep cleanings. Oral pathogens disseminate systemically through the bloodstream, initiating a cascade of physiological responses. In our case report, this systemic spread was initiated by a dental procedure on the right side and triggered the activation of systemic coagulation pathways, leading to thrombus formation as a protective mechanism against pathogen dissemination. Moreover, the presence of circulating pathogens elicited an inflammatory response within the vasculature, termed vascular inflammation, characterized by endothelial dysfunction and immune cell infiltration.

Stroke is a multifactorial disease, influenced by both genetic and environmental element. Well-established risk factors include gender, age, diet, education, physical activity, smoking, alcohol consumption, stress, diabetes, hypertension, and cardiovascular diseases [16]. Additionally, recent epidemiological studies have indicated a potential association between oral infection-associated diseases such as periodontitis and stroke risk [17,18]. In general, there is growing evidence that chronic exposure to infections may elevate stroke risk, with acute infections often serving as direct triggers [19]. Inflammation, a fundamental response of the body to surgery or injury, is pivotal in cardiovascular diseases such as heart attacks and ischemic strokes. This inflammatory response can be triggered by several factors, including bacterial infections or surgical interventions. Notably, poor oral hygiene can initiate a systemic inflammatory response due to the invasion of oral bacteria and inflammatory mediators, significantly increasing the risk of stroke, which has been shown in the case of periodontitis [20]. This connection highlights the importance of maintaining good oral hygiene as part of comprehensive stroke prevention strategies.

Our case uniquely illustrates a direct connection between oral anaerobic bacteremia and stroke following a wisdom tooth extraction, suggesting a pathway through which systemic invasion of oral bacteria can lead to direct vascular inflammation and endothelial dysfunction.

In the context of the case presented here, the identified pathogens—*F. nucleatum*, *P. gingivalis* and *P. micra*—are anaerobic bacteria, often linked with periodontal diseases, and have been implicated in distant site infections, including brain abscesses [21,22,23,24,25,26,27,28,29]. Yet their direct association with ischemic stroke remains rare and underreported. Since the diagnostics of these pathogens is not part of standard diagnostics and was only possible through next-generation sequencing, it is possible that such pathogens often remain undetected. Notably, *P. gingivalis* is capable of evading host immune responses and can circulate through the bloodstream and lymphatic system, colonizing arterial walls and causing direct inflammation within blood vessels, which also leads to the production of systemic inflammatory mediators, playing a substantial role in the pathophysiology of various vascular diseases [30]. Furthermore, if not promptly and adequately addressed, oral pathogens can initiate severe inflammatory responses that spread beyond the oral cavity, potentially impacting other areas of the mouth and causing significant alterations in other organs and tissues, such as rheumatoid arthritis [31].

Interaction of bacteria with eukaryotic hosts including humans is not yet well understood. For establishing an effective infection, bacteria must communicate with each other as well as with their host, a process referred to as quorum sensing and interkingdom signaling, respectively [32,33]. Main pathogenicity factors like toxins as well as biofilm formation are under control of those bacterial cell–cell communication systems, making those communication systems promising targets for antimicrobial treatment. For example, ajoene isolated from garlic has been demonstrated to block bacterial communication of the opportunistic pathogen *Pseudomonas aeruginosa* and, therefore, bacterial adhesion, suggesting this compound as promising therapeutic in cystic fibrosis treatment [34]. It has been demonstrated for several bacteria in periodontopathic biofilms to produce signals for intra- and inter-species communication, among these are *P. gingivalis* and *F. nucleatum* [35]. Although the main communication molecules used for signaling in many bacteria are known, the communication between bacteria and their host is not well understood to date [36]. However, it is known that, e.g., stress hormones like epinephrine and norepinephrine trigger pathogenicity of the gastrointestinal pathogen *Salmonella enterica* serovar Typhimurium, demonstrating that hormones can be important players in interkingdom signaling [37]. Furthermore, it has already been shown that norepinephrine of *F. nucleatum* may promote colorectal cancer via the quorum sensing system of the bacteria [38]. Therefore, it is possible that stress hormones produced by the patient of the case presented in this study after wisdom teeth removal might have triggered the bacterial community to establish infection of the adjacent neurons. This idea is supported by the fact that bacteria of the gut microbiome have been demonstrated to directly interfere with the endocrine system, a field named microbial endocrinology [39]. Since *P. gingivalis* has also been brought into correlation with Alzheimer’s disease [40], it is possible that these bacteria might interact with the human neuronal and the endocrine system. It will therefore be important to focus on the bacterial quorum sensing as well as the interkingdom systems of oral pathogenic bacteria as targets for the development of novel drugs that can be used for treatment as alternatives or in combination with classical antibiotics in the future.

This case highlights the importance of considering bacteremia from oral pathogens as a significant risk factor for vascular events post-dental procedures. The acute onset of neurological symptoms following the extraction, coupled with the identification of specific periodontopathogens in the cerebrospinal fluid (CSF), points towards a pathogenetic sequence where anaerobic oral bacteria may induce systemic vascular events through mechanisms that are not fully understood but likely involve direct bacterial invasion and subsequent inflammatory responses within cerebral vasculature.

Given the potential for significant morbidity associated with such occurrences, it is crucial for dental and medical professionals to maintain a high index of suspicion for bacteremia-induced complications following invasive dental treatments. Preventative measures, including appropriate antibiotic prophylaxis and careful monitoring of patients with predisposing factors for bacteremia, should be considered to mitigate these risks.

The case presented here raises the question of whether prophylactic administration of antibiotics and/or rinsing with a mouthwash solution by the patient can reduce the risk of bacteremia or stroke. Even if bacteremia can be reduced by antibiotics, such administration must be weighed against possible risks, such as anaphylactic shock, gastrointestinal issues, and the development of bacterial resistance. Therefore, there is no recommendation for such prophylactic administration of antibiotics prior to the extraction of wisdom teeth in otherwise systemically healthy patients, as in our case. However, the use of a preoperative mouth rinse by the patient may be an alternative to antibiotic prophylaxis. Nonetheless, there is no solid recommendation based on systematic meta-analyses for preoperative mouth rinsing with antimicrobial agents prior to wisdom tooth extraction. A meta-analysis that examined the effect of a mouth rinse containing chlorhexidine on the reduction of bacteremia found that only 12% of bacteremia cases could be prevented [41]. Further studies are required to clarify whether preoperative mouth rinsing, possibly also with other antimicrobial agents, leads to the same or even better effects, so that a solid clinical recommendation can be made.

Moreover, the development of symptoms suggestive of systemic infection or neurological compromise after dental procedures warrants immediate clinical attention to prevent severe outcomes as illustrated by this case.

In conclusion, while the link between oral bacteremia and ischemic strokes is uncommon, it necessitates awareness and vigilance among healthcare providers to recognize and promptly address this potential complication. This case serves as a reminder of the broader clinical implications of oral health and its management, especially in the context of invasive dental procedures.

## 4. Conclusions

When assessing patients with “cryptic” intracerebral and intraspinal infections, it is crucial to consider the potential contribution of oral infections with bacteremia. Inquiring about any invasive dental procedures performed within the previous four weeks is of utmost importance in the evaluation of such patients. The present case shows, impressively, that an initially uncomplicated wisdom tooth removal can have serious consequences.

## Figures and Tables

**Table 1:** Blood and cerebrospinal fluid tests.

Sample	Examinations	Result
CSF	General bacteriology	Negative No growth of aerobic or anaerobic microorganisms
	data	data
CSF	Mycobacteria	Negative *Mycobacterium tuberculosis* complex: negative
CSF	Syphilis	Negative *Treponema pallidum* antibodies negative
CSF	Borrelia	Negative *Borrelia burgdorferi* IgG & IgM antibody ELISA: negative
CSF	*Listeria monocytogenes*	Negative
CSF	Molecular Biological Diagnostics (16S rRNA gene PCR)	Negative No evidence of bacterial DNA
CSF	Multiplex PCR for Diagnosis of *Mycoplasma pneumoniae/Chlamydia pneumoniae*	Negative Multiplex PCR: negative
CSF	Zygomycetes	Negative Zygomycetes: negative
CSF	*Cryptococcus mycosis*	Negative *Cryptococcus mycosis*: negative
CSF	Molecular Biological Diagnostics of mycotic DNA (18S rRNA gene PCR)	Negative No evidence of mycotic DNA
Blood	General bacteriology (Aerobic and anaerobic blood cultures)	Negative No growth of bacteria and sprout fungi
Blood	Tuberculosis Quantiferon Set	Positive QuantiFERON-TB Gold
Blood	Zygomycetes Diagnosis of Brucellosis	Negative No serological evidence of infection
Blood	Beta-d-Glucan for Diagnosis of Pneumocystis Pneumonia	Negative No indication of an invasive mycosis or pneumonia caused by *Pneumocystis jirovecii*

**Table 2:** Extensive genetic diagnostics (Noscendo Pathogen Diagnostics; Noscendo GmbH).

Sample	Examinations	Result
CSF & Blood	NGS (Noscendo GmbH)	Positive for: *Fusobacterium nucleatum*, *Porphyromonas gingivalis*, *Tannerella forsythia*, *Parvimonas micra*, *Campylobacter showae*
Biopsy TMJ right	NGS (Noscendo GmbH)	Negative No growth of bacteria or fungi
Biopsy Musculus pterygoideus lateralis right	NGS (Noscendo GmbH)	Positive for: *Saccharomyces cerevisiae* *Porphyromonas gingivalis*, *Desulformicrobium orale* *Fusobacterium nucleatum*, *Bacilus cereus*, *Tannerella forsythia*, *Pseudobacterium alactolyticus* *Entarnoeba histolytica*, *Escherichia coli* *Dialister pneumosintes* *Aspergillus carbonarius* *Parvimonas micra*,

TMJ = Temporomandibular joint.

## Data Availability

The detailed presented data are available upon request from the corresponding author. The data are not publicly available due to our hospital data privacy policy.
